# QoS-Aware Approximate Query Processing for Smart Cities Spatial Data Streams

**DOI:** 10.3390/s21124160

**Published:** 2021-06-17

**Authors:** Isam Mashhour Al Jawarneh, Paolo Bellavista, Antonio Corradi, Luca Foschini, Rebecca Montanari

**Affiliations:** Dipartimento di Informatica—Scienza e Ingegneria, University of Bologna, Viale del Risorgimento 2, 40136 Bologna, Italy; paolo.bellavista@unibo.it (P.B.); antonio.corradi@unibo.it (A.C.); luca.foschini@unibo.it (L.F.); rebecca.montanari@unibo.it (R.M.)

**Keywords:** mobility data, Apache Spark, approximate query processing, spatial data, Internet of Things, sampling, continuous queries

## Abstract

Large amounts of georeferenced data streams arrive daily to stream processing systems. This is attributable to the overabundance of affordable IoT devices. In addition, interested practitioners desire to exploit Internet of Things (IoT) data streams for strategic decision-making purposes. However, mobility data are highly skewed and their arrival rates fluctuate. This nature poses an extra challenge on data stream processing systems, which are required in order to achieve pre-specified latency and accuracy goals. In this paper, we propose ApproxSSPS, which is a system for approximate processing of geo-referenced mobility data, at scale with quality of service guarantees. We focus on stateful aggregations (e.g., means, counts) and top-N queries. ApproxSSPS features a controller that interactively learns the latency statistics and calculates proper sampling rates to meet latency or/and accuracy targets. An overarching trait of ApproxSSPS is its ability to strike a plausible balance between latency and accuracy targets. We evaluate ApproxSSPS on Apache Spark Structured Streaming with real mobility data. We also compared ApproxSSPS against a state-of-the-art online adaptive processing system. Our extensive experiments prove that ApproxSSPS can fulfill latency and accuracy targets with varying sets of parameter configurations and load intensities (i.e., transient peaks in data loads versus slow arriving streams). Moreover, our results show that ApproxSSPS outperforms the baseline counterpart by significant magnitudes. In short, ApproxSSPS is a novel spatial data stream processing system that can deliver real accurate results in a timely manner, by dynamically specifying the limits on data samples.

## 1. Introduction

Large amounts of geo-referenced data streams are generated daily from IoT devices in high-traffic dynamic smart cities [1]. The data arrive in streams, characterized as highly skewed in data patterns and distributions [2]. For example, concentrations of vehicular and human mobility data differ in density during various periods of the week. Novel initiatives, motivated by such an explosion of data availability, have been introduced in smart cities to exploit such data in building real-time applications that benefit citizens in many aspects. For example, meteorological data are joined with vehicle mobility data in metropolitan cities, so that municipalities can distinguish areas with more vehicle-causing air-pollutants, such as particulate matters (PM2.5 and PM10). Such dynamic smart city applications are only made possible because of the abundance of various technologies that operate synergistically to achieve such applications, including Cloud, Edge, and Fog computing, working on big data coming from the Internet of Things (IoT) [3]. The realization of a smart city vision involves three main components: data collection by using IoT devices, intercommunication among IoT instruments, storage, and processing deployments (such as cloud computing), and front-end exploitation systems that are able to analyze and visualize data (to help make strategic decisions regarding city operations and infrastructure). Various smart cities have been designed worldwide; however, few remain sustainable. This is so because the size and variety of data are changing at a pace that far exceeds pre-planned IT infrastructure and capacity.

Those initiatives require data management systems to deal with the large amounts of data that arrive endlessly from billions of IoT devices. Data are typically geo-referenced (i.e., have spatial dimension, such as meteorological, mobility, and microblogging data). Maintaining system stability while dealing with such high volumes of spatial data, for supporting time-sensitive real-time applications, remains a challenge that overcomes the capabilities of current offline big data management systems.

The characteristics of IoT data arriving in fast streams, and the aforementioned requirements, require more proactive approaches to process unbounded data streams, while arriving continuously in real-time. To provide timely insightful analytics, stream processing engines (SPEs), such as Apache Spark [4] and Apache Flink [5], employ auto-scaling by overproviding extra cloud computing resources to achieve latency goals and prevent system crashes. They also apply backpressure, which pushes back the lateness to ingestion systems and, thus, limits the size of data that can arrive to the SPE operator downstream the system. However, this may lead to inaccurate real-time results [6]; in addition, those systems do not feature quality of service (QoS) control for geo-referenced data streams [2,7].

In this paper, we focus on answering spatial queries with QoS guarantees. For example, how our system would be able to grant an error bound that does not exceed a certain threshold, while periodically computing the average speed of vehicles, every minute, given the uncertainty in data arrival rates and skewness. To answer such an interactive spatial query, an online procedure needs to compute an approximation for the minimum data size (i.e., sample size) that would be required for every time window (e.g., one minute) to remain within the pre-specified error bound. Moreover, because of the temporal variation in data arrival rates and skewness, error and accuracy control components should operate over in-transient data streams in real-time. In other terms, we seek to answer the question on: how to improve current SPEs, so that we harness their power in achieving low latency and/or high accuracy while serving fresh IoT smart city data to exploration applications (e.g., maps and visualizations). To achieve this goal, the approach proposed in this paper focuses on exploiting data stream processing over continuously arriving data coming from nonstationary IoT devices in urban metropolitan cities. The main task involves stateful aggregations, where summaries are generated and incrementalized stepwise for additional analysis. We specifically propose a novel system, which we term Approximate Spatial Stream Processing System (ApproxSSPS), for quality, approximate processing of geo-referenced data streams coming from dynamically moving IoT devices. We specifically focus on achieving pre-specified latency and/or accuracy goals. ApproxSSPS computes approximate spatial statistics (e.g., average of a target variable, such as ‘the average speed of cars’) gradually, over fast-arriving spatial data streams. It is also able to perform Top-N queries at scale. This design choice is motivated by the fact that stock versions of current SPEs from the literature are unable to maintain stability due to the fluctuations in the size and speed of data arriving from IoT devices in dynamic smart cities. Resorting to quality control, as per our novel system ApproxSSPS, helps applications remain responsive during peak loads at the cost of tiny permissible accuracy. Moreover, ApproxSSPS embraces a general-purpose architecture that enables testing the reliability and scalability of current big data management technologies in supporting time-critical smart city scenarios.

Our system is composed of five components: monitor, statistics aggregator, sampler, controller, and approximator. The monitor actively senses the stream for fluctuations in data arrival rates, then it informs the controller in cases where the arrival rate exceeds the processing capacity downstream. The sampler employs state-of-the-art online spatial sampling methods (from our previous works, either SAOS [2] or ex-SAOS [7]) to sample representative geo-referenced data during transient peaks in data arrival rates. The statistics aggregator collects real-time performance data and serves them to the controller. The controller computes sample limits that achieve latency or/and accuracy goals, which are pre-specified in the user query. The approximator is responsible for receiving the sampled data and generating an answer for the spatial query.

In summary, the following are our contributions in this paper (1) we designed an accuracy/latency aware data stream processing system, ApproxSSPS, for statistics computations and aggregation queries of mobility data in dynamic smart cities. ApproxSSPS contains accuracy and latency controllers for maintaining system stability during transient peaks in data arrival rates; (2) we compared ApproxSSPS with a similar baseline system from the recent literature, specifically, we compared it with the work by [8]; (3) we implemented ApproxSSPE on Spark Structured Streaming and evaluated it using real geo-referenced big mobility data streams. ApproxSSPS can analyze mobility data at scale with QoS guarantees, in a way that outperforms the baseline by significant magnitudes. Our results show that, by deploying our approach on cloud computing resources, we obtain gains in achieving QoS goals (i.e., lowering latency and increasing accuracy) that significantly outperform the baselines from the current state-of-the-art.

The paper is organized as follows. Section 2 establishes relevant foundations that will guide the discussion thereafter; it also reviews the relevant related literature. Section 3 shows, in detail, the design of ApproxSSPS, with the peculiarities and characteristics it provides for approximate query processing in spatially rich environments. Section 4 discusses a similar system from the relevant recent literature (which we refer to as the baseline). In the same section, we show the results and discuss the performance of ApproxSSPS against the baseline. In Section 5, we conclude the article and discuss limitations in addition to potential future research works.

## 2. Related Work

Decision makers in smart cities are interested in information that shows how vehicle mobility patterns affect traffic. Mobility traces are geo-referenced data that arrive very fast, in streams, and are mostly skewed in distribution [2,7]. Data stream processing systems aim to achieve time-based and accuracy-based QoS goals while answering such queries. Those goals are conflicting and achieving both is intractable. More accuracy requires more data, while less latency requires less data. Systems seek to strike a plausible tradeoff between those QoS goals. The system described in this paper was specifically designed to achieve those requirements. This section introduces state-of-the-art solutions from the current literature, which deal with approximate query processing and data management quality control in the context of smart cities and IoT.

### 2.1. Methods for Resolving Online Information Overloading

In dynamic smart cities, huge amounts of geo-referenced data streams arrive at cloud-based SPEs [9], sometimes at unprecedented rates that far outpace their capacities, rendering them irresponsive at peak hours [2,7,8]. To maintain system stability, stock versions of current SPEs respond to those fluctuations with various strategies, including the following: adaptivity and elasticity. A strategy that is known as backpressure is one form of adaptivity. SPEs that feature backpressure work on system stability by trading-off accuracy for latency gains. That said, they sense the data stream and project it with the system capacity. If it far exceeds the system’s capacity, they send an order to the ingestion layer upstream, forcing it to lower the rate of tuples it sends downstream. This pushback mechanism, however, causes a significant loss in accuracy during spikes in data arrival rates. Moreover, most interesting mobility data analytics happen during those transient spikes. Losing real-time by forcing backpressure will then lead to the inability of the system to get insight during those periods. Approximate query processing (AQP) is another form of adaptivity. Sampling is a common method in AQP that works by selecting miniscule data in a way that only leaves statistically insignificant rigorous error bounds, in return for a statistically significant latency gain. AQP is a plausible solution because decision makers in smart cities are willing to accept tiny losses in accuracy in exchange for significant speedups, from ingestion to insights [2,7]. The difference between AQP and backpressure is that the former does not stop data temporarily from arriving downstream. It otherwise selects a representative sample that only affects the accuracy in acceptable margins.

Elasticity techniques work by regularly provisioning and de-provisioning resources dynamically based on the data arrival rates [10,11]. This strategy however imposes extra overhead caused by the continuous re-configurations of cloud resources while the system is busy working with online data.

The plain versions of those QoS-aware solutions are unaware of spatial data characteristics. Geo-referenced data arriving from moving objects are parametrized and represented as coordinates (normally longitudes/latitudes). This means that spatial data lose their shape while in-transit and reconstructing it to project it back to the original shape in real geometry is an expensive spatial join [12,13], or, stated in another way, specifying to which area in real geometry each parametrized pair belongs. For an SPE to operate on mobility data efficiently at scale, it should incorporate components that are aware of the spatial characteristics of data. In addition, it should react appropriately to sudden spikes in data arrival rates in a way that maintains the system stability. However, online spatial sampling is not an easy matter due to the multidimensional shape of mobility data. Current versions of SPEs cannot achieve QoS and spatial awareness intrinsically altogether [2,7,13,14]. To achieve those goals, we have designed ApproxSSPS. As it features the two state-of-the-art online sampling methods from our previous works (SAOS and ex-SAOS) in the sampler component, we first review those methods before delving into the peculiarities of ApproxSSPS in Section 3.

### 2.2. Online Spatial Data Sampling

SAOS [2] and ex-SAOS [7] are two state-of-the-art online spatial data sampling methods for cloud-based SPEs. They both work pretty much the same way, but with different levels of granularity. They both start by computing the geohash covering of every neighborhood in a city (i.e., municipality administrative divisions). Geohash is a spatial indexing method that generates a string representing all points confined within a rectangular area.

**Figure 1:**
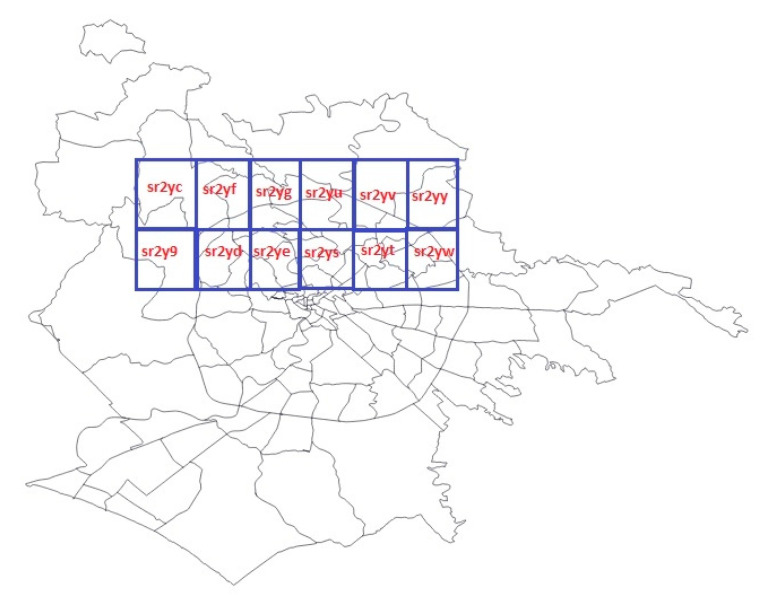
Partial view of the geohash covering near the city center of Rome in Italy. Geohash precision is 25 (i.e., 5 characters). Mobility data traces that are nearby by certain distance are enclosed within the boundaries of the same geohash area; thus, have the same geohash index value. Several geohash values cover a specific neighborhood. This is known as the geohash covering. Edge cases occur in the situation where several points share the same geohash value despite belonging to different neighborhoods. Those cases are resolved by applying the spatial join operator.

To take a utilitarian perspective, consider the Earth as a planar geometry flattened out to two-dimensions and a grid with regularly-sized cells overlay that geometry (as shown in Figure 1); geohashes are strings generated in such a way that geometrically-nearby spatial objects share the same geohash value prefix [14]. For example, several objects near the city center of Rome, Italy, have the geohash code that is equal to ‘sr2yk’, which is a geohash that has a length that equals 5. This means that it covers a rectangular area (cell), which has a width that roughly equals 4.89 km, and a height that also equals 4.89 km. The longer the geohash length, the higher the accuracy of the granular cell it is representing. For example, a geohash with a length that equals 6 represents a cell with a width that roughly equals 1.22 km and a height that equals 0.61 km. The longer the shared prefix, the more proximate the spatial points can be found in real geometries.

Having generated the geohash covering of the city, SAOS proceeds as it follows. It selects fair count of geo-referenced data stream tuples from each division (grid cell) individually. That way, it selects the same proportion of spatial points from each cell individually. This is equivalent to selecting a percentage of points that belong to each geohash. Each administrative area in the city (known as neighborhoods) is represented by a polygon, as shown in Figure 1, where a set of polygons divide the administrative parts of Rome. Few geohashes then constitute the covering of each neighborhood of the city. Selecting a specific percentage of points from each group of points represented by a geohash, we are selecting, approximately, the same proportion (percentage, that is the sampling fraction or rate, a configurable parameter in the SAOS algorithm) of spatial points from each neighborhood independently. This design is a spatial analogy to the stratified sampling. As much as the words can be conceivable, SAOS acts as a tessellation method in a two-dimensional space. However, a number of the so-called edge cases appear that way. This is so, because geohash indexing is an approximation, which means that few tuples have the same geohash while belonging to neighborhoods that are far apart. To improve SAOS, in a previous work, we designed ex-SAOS [7], so that it works on a coarser level. It resolves the edge cases by applying a well-performing spatial join method, so that it determines which neighborhoods each edge case belongs. It thereafter samples the same exact proportion of points from each neighborhood individually.

### 2.3. QoS-Aware Big Data Management

The closest system in the related literature that employs custom methods for QoS control during data management is a system introduced by [8]. They designed an architecture that incorporates a weight-based stratified-like sampling method. It resembles a binary tree for dividing the arrival data streams into pairs of buckets, based on local maximum and minimum values of the attributes that need to be estimated (e.g., average speed in mobility data). They also injected an error controller and sampling fraction estimation module. They apply the error-control model to adjust the weights of each stratum (node in the binary tree as per their design) to minimize the error. However, they do not provide direct support for spatial data stream approximate processing. Attempting to apply their design to highly skewed spatial data will normally result in over-flooding a few buckets, while others are empty, thus, deteriorating the system performance.

In addition, EXPLORA [15] is an approximate processing system designed for supporting approximate visualization of spatiotemporal data, with quality of service guarantees. It features an online synopsis method that interactively collects representative samples of data, aiming to achieve high throughput, while minimizing accuracy loss. However, the system does not feature online controllers to keep up with the pace of the online spatial data arrival rates.

Moreover, SnappyData [16] is a system that is engineered atop Apache Spark, and features AQP for general data stream workloads. It employs probabilistic data structures, aiming to fulfill accuracy and/or latency targets. However, it is not tuned to the multidimensional properties of IoT geo-referenced data. Hence, it does not employ a spatial-aware sampler and, thus, is not able to select spatial representative data from IoT data.

In addition, Taster [17] features the following main components: synopses collector and warehouse, cost-based planner, and tuner. The cost-based planner interactively generates a set of approximate execution plans. Those plans are applied on synopsis (sketches or samples), computed either online or pre-stored in a synopsis warehouse (for recurrent exploitation in case of similar queries). All of those plans should fulfill the accuracy targets pre-specified by the user. The cost of each plan is then served to the tuner, which selects the best plan in terms of the performance gain. Then, the plan is applied to collect the synopsis (persisted offline or computed on the fly). Approximate results are then computed based on those synopses to obtain an answer for the user query. However, this framework suffers from three shortcomings. It is untuned for spatial data. Moreover, it keeps the persisting synopsis in a warehouse, which adds extra I/O overhead to the system. Moreover, it does not incorporate a spatial-aware approximator that enables approximations on geo-referenced data.

Moreover, ApproxHadoop [18] employs a two-stage sampling method for Hadoop to drop data on complete tasks or tuples levels, aimed at reducing the data access overhead. However, it depends on an offline profiling mechanism for tuning the accuracy/latency levels. It is then inappropriate for data stream settings.

Sampling is also utilized for other spatial workloads, such as spatial partitioning, as it appears in Simba [19] and SpatialHadoop [20]. However, those systems do not include components for controlling the sampling rate based on user pre-specified latency/accuracy targets. Moreover, they are not suitable for online stream processing of geo-referenced data.

## 3. ApproxSSPS: Approximate Processing of Spatial Data Streams in Smart Cities

In the previous section, we discussed current approaches that deal with big data management at transient times, when data arrives in fast streams that exceed system processing capacity. Most of those systems handle the problem from a general aspect that ignores the spatial dimension of the data. Other systems handle spatial disk-resident data and, therefore, are unable to deal with data streams.

The proposed system in this paper builds atop those systems and introduces a general-purpose system, termed ApproxSSPS. This stream processing system accepts the accuracy and/or latency as QoS goals expressed within a continuous spatial query. Thereafter, it employs state-of-the-art spatial sampling methods from our previous works (SAOS [2] and ex-SAOS [7]) to limit the number of tuples that arrive at a spatial approximation downstream. It does so with QoS guarantees, in terms of latency or/and accuracy expressed within the query. Our system aims to speed up online spatial queries in support of interactive analytics, in the context of smart cities. In this section, we discuss the system components and architecture. We then explain the main features and requirements involved in designing the system architecture.

### 3.1. System Architecture and Features

**Figure 2:**
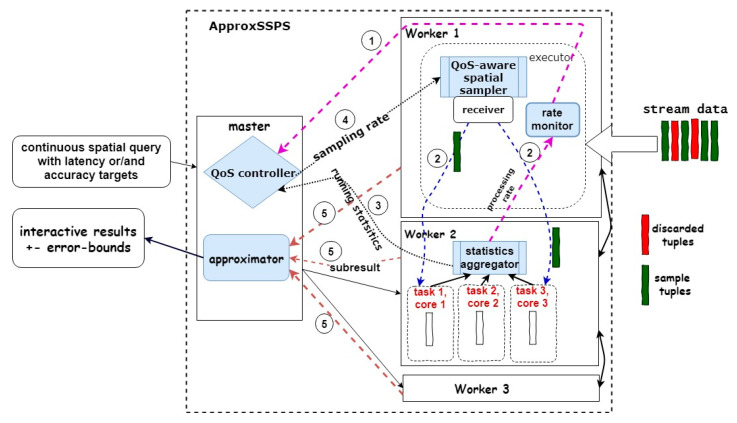
Overview of ApproxSSPS architecture incorporated with Spark Structured Streaming. Components of ApproxSSPS are highlighted in light blue. Numbers in the circles show the sequence at which the system operates, from the point it receives continuous data streams at one of the worker nodes, passing through the QoS sampler. Thereafter, samples are distributed to the other worker nodes where they are processed continuously. Running statistics are regularly fed to the QoS controller, which decides upon the allowed sampling rate and feeds it back to the sampler. Sub results are accumulated from all worker nodes and sent to the approximator that is responsible for computing an interactive result stepwise and serve it to the user with the error bounds.

ApproxSSPS consists of five components: *monitor*, *statistics aggregator*, *sampler*, *controller*, and *approximator*, as shown in Figure 2. The rate *monitor* is periodically loaded with the average processing rate of the ‘*statistics aggregators*’. If it notices a high spike in the arrival rate that exceeds the processing rate, it sends a signal to the *controller*. The controller also receives a signal from the statistics aggregators serving the average processing rate in the last few time intervals (batch intervals in SPEs terms) for each worker machine. The *controller* then calculates the appropriate sampling rate that guarantees achieving QoS goals (either latency or accuracy) pre-specified within a continuous spatial query. This sample rate is then served to the *sampler* in the front-stage, which then employs either SAOS [2] or ex-SAOS [7] state-of-the-art online spatial data sampling methods to sample the desired size from the arriving data stream. The data sample is then divided to the tasks in each worker node. Each task computes a sub-result for answering the query and serves it to the *approximator* in the master machine. The approximator then combines the sub-results and generates an incremental answer for the continuous spatial query. It thereafter serves the stepwise result interactively to the user in the presentation layer (i.e., for visualization, dashboarding, etc.), together with the corresponding error bounds. Figure 2 shows how we incorporated those new components with Spark Structured Streaming [21] (a variant of Spark Streaming [22] with an SQL-like support). The spatial sampler (either SAOS or ex-SAOS) is a new layer that is incorporated atop Spark’s plain receiver. An example continuous spatial query is a stateful aggregation that shows the “counts of mobility tuples in each neighborhood of a city” incrementally. We featured the statistics aggregator within Spark Structured Streaming in a way that enables it to collect statistics periodically (every Spark’s job interval) and serve it to the controller and the approximator. Latency or/and accuracy targets are served to the system by the user as part of a continuous spatial query. As the QoS controller constitutes a pivotal component in ApproxSSPS, we comprehensively discuss it in Section 3.2. Incorporating QoS-awareness in this way guarantees that the system remains stable without overburdening developers with the task of handling these logistics manually.

### 3.2. QoS Controller Component of the System

It is the responsibility of the QoS controller to compute the appropriate sampling rate to fulfill latency or/and accuracy QoS goals. Itis comprised of two parts: a controller that measures the latency imposed in the latest few job intervals, in addition to a controller that measures the accuracy based on the theory of statistics. We decided to achieve either a lower latency or more accuracy as specified in the continuous spatial query. However, the system is able to achieve a plausible balance between them. In this subsection, we describe how the controller achieves latency or/and accuracy QoS goals by informing the decision on the data size that the sampler needs to drop.

#### 3.2.1. Accuracy Controller

The accuracy controller employs a well-established equation from the theory of statistics for calculating the number of data stream tuples that are allowed to arrive at the Spark’s plain receiver for the next Spark’s job interval. We specifically employ Equation (1) adapted from [23].

(1) n=(Zα2)2 (varε2)

where n is the number of tuples that may be allowed down the road towards the operators of the approximator. In a standard normal distribution, z_α/2_ is the (1 − α/2)^th^ percentile of the standard normal distribution [23]. For a 95% confidence level (confidence level=100(1−α)), we have z_α/2_ = 1.96. var is the variance of the target variable. Table 1 shows the most common z_α/2_ values. That is to say that, for a 95% confidence level, which is to be served as part of the spatial continuous query, Equation (1) resorts to Equation (2).

(2) $$n=3.84\times \left(\mathrm{var}/\varepsilon^{2}\right)$$

where ε is the margin-of-error. It is measured as half the width of a 95% confidence interval. Common value is 0.03, which means that with a 95% confidence level, the sampling would result in an estimation for the target variable (e.g., average speed in taxicab mobility data) that is only ∓0.03 far from the real value.

As contrary as it may seem, even though mobility data from IoT in smart cities are highly skewed, statistics on target variables in such data have shown normal distribution (informally, a bell-shaped curve) in sampling distribution. Thus, in accordance with the Central Limit Theorem (CLT) [23], traditional statistics still apply, even though spatial data streams normally arrive in unpredictable bursts. CLT dictates that the probability distribution converges to a normal distribution while the sample size increases. We empirically found that, for our data, which we used for testing, despite the original population data being highly skewed, a sample of 1000 ‘*means*’ of values converges to a normal distribution.

#### 3.2.2. Latency Controller

The latency controller is responsible for sensing a staggered latency that accumulates because of transient bursts in data arrival rates, which exceed capacities of computing resources downstream in the worker machines. We employ a simple, yet efficient, model-based approach. We simply rely on Equation (3).

(3) $$rate_{new}=rate_{avg}-\left(\alpha .e\right)$$

where $rate_{new}$ is the new data rate that is allowed to arrive at the SPE operator downstream, to account for the excess amount of arriving tuples that have caused the latency. $rate_{avg}$ is the average processing rate during the last few batch intervals, whereas α is a regulation factor (a scalar value between 0 and 1). $rate_{avg}$ is only calculated from the latest few batch intervals when there are no significant delays observed. Moreover, *e* is the staggered error that accumulates in the last few batch intervals, which is calculated by Equation (4).

(4) e=(delay×rate)time

*rate* is then the current processing rate. *time* is the ‘batch interval’, whereas *delay* is the task scheduling delay that is induced by the excess rate of arriving stream tuples.

Equation (3) then resorts to the following conceptualization: by the second term (α × *e*), we calculate an error that results during the last few batch intervals. This is related to the number of tuples that were not processed for the lack of computing capacity in the worker nodes. Deducting that from the average stable observed processing rate during the latest stable batch intervals, we then obtain a good estimate for the new rate that is allowed in the system for the next batch interval. This will bring the system back into stability. That rate is served back to the spatial sampler in the front stage (either SAOS or ex-SAOS) to limit the number of tuples that will be sent to the receiver. In Equation (3), α constitutes a *regulation factor* that dictates how much we want to account for the staggered delay caused by excess arriving tuples in the last few batch intervals. For example, ‘1’ means that we are fully aware of the historical error. The lower the α, the less we account for the staggered accumulated tuples caused by the excess amount of data. Equation (4) is analogous to the formulation that specifies how much more tuples could be processed if there was no such delay.

Delay can also be negative if the processing rate is faster than the arrival rate. For example, consider a batch interval that equals 1000 milliseconds, the number of the arriving tuples is 80 K per second, and the processing capacity of the system is 100 K, meaning that 800 milliseconds is used for processing, while the system remains idle for the remaining 200 milliseconds. In this case, the delay is negative (i.e., −200), which means that the next allowed rate will increase, allowing more tuples to be ingested to exploit the full processing capacity of the system. In this case, also, the regulation factor α plays a pivotal rule. In other terms, if we want to be fully attentive for possible future spikes in data arrival rates, we keep α lower than one, even when the system processing capacity is permissive of 3.3 metrics.

We show the statistics used to calculate the estimations of target variables (e.g., average speed) in the approximator component.

ApproxSSPS supports linear queries (using the approximator) that estimate geo-statistics for interesting target variables. For example, “what is the average speed of taxis in each district of a city”.

To this end, we apply (5) to compute the estimated average.

(5) $${\bar{Y}}_{\mathrm{str}}={\hat{t}}_{\mathrm{str}}/N=\overset{I}{\underset{i=1}{\sum }}(N_{i}/N){\bar{y}}_{i}$$

where ${\hat{t}}_{\mathrm{str}}=\overset{K}{\underset{k=1}{\sum }}t_{k}=\overset{K}{\underset{k=1}{\sum }}N_{k}{\bar{y}}_{k}$.

Since those queries are based on samples instead of the population, they are tied to specific degrees of uncertainties, which need to be measured to validate the correctness and efficiency of the sampling methods. We applied the same set of uncertainty quantification metrics for both SAOS and ex-SAOS [2,7]. In other terms, to quantify for linear queries, we apply (6).

(6) $$SE({\bar{Y}}_{\mathrm{str}})=\sqrt{\hat{v}({\bar{y}}_{\mathrm{str}})}$$

where $SE({\bar{Y}}_{\mathrm{str}})$ is the standard error that results from estimating the target variable by depending on the sample instead of the population. To calculate the standard error of a mean (average) estimator for a target variable, the estimated variance is computed using (7).

(7) $$\hat{v}({\bar{y}}_{\mathrm{str}})=\hat{v}({\hat{t}}_{\mathrm{str}})/N^{2}$$

where $\hat{v}({\bar{y}}_{\mathrm{str}})$ is the estimated variance of the estimated average, whereas $\hat{v}({\hat{t}}_{\mathrm{str}})$ is the estimated variance of the estimated total. N is the accumulated total number of tuples arrived up to that moment in time. We compute $\hat{v}({\hat{t}}_{\mathrm{str}})$ using (8).

(8) $$\hat{v}(\hat{t_{\mathrm{str}}})=\sum_{k=1}^{K}(N_{k}-n_{k}/N_{k})(N_{k}^{2}s_{k}^{2}/n_{k})$$

$N_{k}$ is the total number of tuples that belong to each stratum (without sampling), while $n_{k}$ is the sample size in each stratum. $s_{k}^{2}$ is the variance of the target variable in each stratum.

Equation (9) shows the relationship between the SE and margin-of-error (which is used in the accuracy controller).

(9) ε=Zα2×SE

where ε is the margin-of-error (i.e., the error bound in the accuracy controller). The proportional relationship shows that the margin-of-error increases linearly as the SE increases. While a more permissive margin-of-error would result in an increased standard error, which means allowing a bigger sample size. ε is half the width of a confidence interval.

## 4. Experimental Evaluation

In the previous section, we showed the architecture and features of ApproxSSPS. In addition, we showed how we implemented its prototype as a proof-of-concept atop Spark structured Streaming, so that it operates in cloud deployments for serving fresh data summaries from IoT data in dynamic smart cities. In this section, we evaluate the effectiveness of ApproxSSPS with real-world geo-referenced mobility data coming from IoT devices in smart cities. We evaluate the performance on single spatial queries. For example, ‘what is the average speed of taxicab data in a metropolitan city?’ We compare the performance of our system, ApproxSSPS, against the baseline system that is discussed in Section 4.1. We begin by describing the baseline system with which we compare. Thereafter, we provide a short description of the testbed and deployment configurations that we use for experimentation. Thereafter, we show the efficiency of ApproxSSPS in terms of its ability to achieve the latency and/or accuracy QoS goals. Moreover, we measure its ability to preserve the stability of the system with the oscillation and fluctuation in data arrival rates.

### 4.1. Baseline System

To show how our novel system, ApproxSSPS, is unique with its overarching traits, as compared to the recent state-of-the-art system, we compare it with a related system. Specifically, we compare ApproxSSPS with the work of [8].

#### 4.1.1. Baseline System Architecture

The closest system in the recent literature that provides similar functionalities for processing data streams with QoS guarantees is presented in the work by [8]. They designed a general approximate processing framework for online data streams. Their framework features three main components: data learning, dynamic sampling strategy, and error control.

Their sampling method design is stratified-like, and they apply a stratification method that is based on binary trees. Their method operates as follows: it first computes the maximum and minimum values of the target variable to be estimated. Thereafter, they split the arriving data stream tuples into the nodes of a binary tree (nodes resemble partitions in data parallel processing systems). Then, for each node, they split again based on the local minimum and maximum values residing in that node. They finish the stratification and, consequently, the splitting of the tree nodes once specific thresholds in the accuracy are reached. As a result, leaf nodes are the strata.

The framework also features an error control component based on the feedback loop mechanism. Based on an error value that is computed at each time window interval, values of weights for each stratum are adapted to minimize the error.

There are three basic shortcomings with this framework. First, the stratification method described is computationally expensive, and adds unnecessary extra overheads as it keeps modifying the splits and traversing the binary tree—also in reverse order—as a maintenance step. Second, the framework, in its stock version, is general and does not have intrinsic support for data with spatial characteristics (i.e., geo-referenced mobility data). The consequence of this is that the stratification method applied is unaware of the spatial co-locality of data. Moreover, it is unaware of the spatial relationships between nearby objects in real geometries. This results in a lopsided division, where spatial objects that are close-by in real geometries end up in different strata, which requires costly data shuffling while processing the data. Preserving co-locality of geo-referenced data has shown superiority in processing performance [9,12,24,25].

#### 4.1.2. Query Performance Metrics of the Baseline System

To compare our system, ApproxSSPS, with the baseline described in this section, we recapitulate the metrics for measuring the statistical efficiency of the baseline system.

The authors of the baseline system [8] designed a binary tree-based stratification strategy that depends on updating strata weights regularly, based on the decision of an error-control module. In this case, the theory of stratification applies [23]. Since their method depends on assigning weights for each stratum, computing an estimate for the average of a target variable requires applying Equation (10).

(10) $${\hat{y}}_{\mathrm{str}}=\overset{H}{\underset{h=1}{\sum }}\;\underset{j\in S_{h}}{\sum }w_{\mathrm{hj}}y_{\mathrm{hj}}/\overset{H}{\underset{h=1}{\sum }}\underset{j\in S_{h}}{\sum }w_{\mathrm{hj}}$$

The variance of the estimated mean is thus calculated by Equation (11)

(11) $$\mathrm{Var}\left({\hat{y}}_{\mathrm{str}}\right)=\overset{H}{\underset{h=1}{\sum }}W_{h}^{2}\sigma_{h}^{2}/n_{h}$$

The standard error (SE) can then be calculated by Equation (12)

(12) $$\mathrm{SE}\left({\bar{Y}}_{\mathrm{str}}\right)=\sqrt{\hat{v}\left({\bar{Y}}_{\mathrm{str}}\right)}$$

Their method applies the Hoeffding inequality [26] (shown in Equation (13)) for calculating the optimal sample size when given an error bound as part of the query.

(13) n≤ln(α2)·(−(b−a)2)2ε2

where a and b are minimum and maximum bounds of values of the target variable, respectively. ε is the error bound that is received as a user input. α is the level of significance (i.e., error making probability upper bound) for a confidence interval surrounding the true value of the estimated target variable (i.e., the true mean in this case) with 2ε.

We adapted the baseline system so that it works with spatial data in cloud deployments. In the next section, we show how our novel system, ApproxSSPS, outperforms the baseline system that is described in this section by statistically plausible margins.

### 4.2. Experimental Environment Setup

Dataset. We used two geo-referenced datasets. The first dataset comes from New York City taxicab trip datasets (https://www1.nyc.gov/site/tlc/about/tlc-trip-record-data.page, accessed on: 5 January 2021), consisting of around 1,400,000 tuples, representing data taxi rides for the first month of 2016. We selected the green taxi trip records, which included fields such as GPS locations and itinerary distances. The target variable in these data is the ‘Trip-distance’, where we aimed to “calculate the average ‘Trip-distance’” and improve the result incrementally as more data arrive.

The other dataset consists of 1,155,654 tuples, representing electric taxi GPS mobility trips for a day in the Chinese city of Shenzhen [27]. The target variable in these data are the ‘speed’. We aimed to calculate the average speed.

Deployment and experimental settings. We ran our tests via a Microsoft Azure virtual network, hosting two directly communicating clusters. The first cluster was an HDInsight cluster hosting Apache Spark version 2.2.0. It consisted of nine NODES (two head nodes + four worker nodes + three zookeeper nodes). Head nodes are of type D12 v2 (with four Cores, 28 GB RAM). Worker nodes are of type D13 v2 (with eight Cores, 56 GB RAM). Zookeeper nodes are of type A2 v2 (with two Cores, 4 GB RAM). The other cluster was an HDInsight Kafka cluster, also having nine NODES (two head nodes + four worker nodes + three zookeeper nodes), with the same capacity characteristics as those for the Spark cluster. Data were stored in CSV files in a BLOB container in the Spark HDInsight cluster. Thereafter, we replayed the data from the CSV files and wrote it to Kafka topics as streams. Apache Spark interactive Jupyter notebooks then read the Kafka data streams and applied ApproxSSPS to find an answer for an interactive spatial query (geo-statistics, such as average speed, or Top-N) with QoS guarantees.

### 4.3. Experimental Results

To measure the performance of our system ApproxSSPS, we adopted a strategy where we measured the two basic capabilities. We first showed the effectiveness of ApproxSSPS from a latency-awareness perspective. We then showed its ability in achieving plausible accuracy–awareness as opposed to those achieved by the baseline system [8].

#### 4.3.1. Latency Controller Ability to Maintain System Stability during Peak Times

We showed the effectiveness of the latency controller of ApproxSSPS in fulfilling latency QoS goals while preserving the stability of the system during transient spikes in data arrival rates. We depended on a scenario where the spatial query requests ‘computed the incremental average of speed for mobility data from Shenzhen city in China’.

We relied on two data-arrival oscillation scenarios: a persistent increase in the arrival rate; and a sudden temporal increase followed by a decrease in arrival rate.

**Figure 3:**
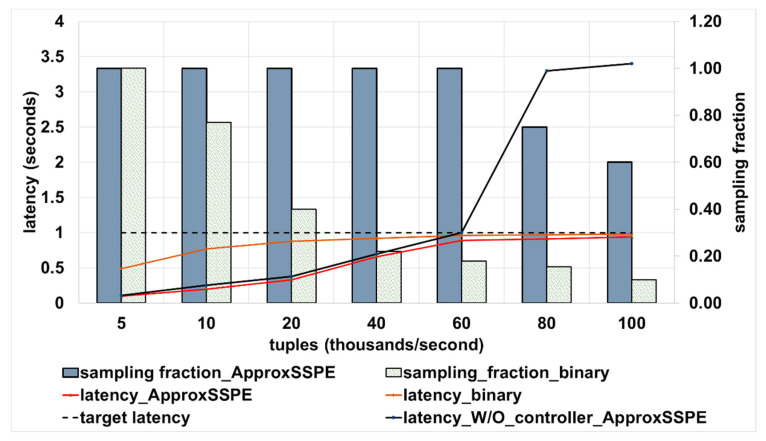
Results of applying latency controller of ApproxSSPS for temporally variable arrival rates. Compared to the binary tree-based baseline [8], and to the case where no controller is applied. A higher sampling fraction is more desirable (i.e., achieves higher accuracy). To maintain system stability and remain within the boundaries of the target latency as more data arrives, where both methods resort to lowering the sampling fraction. However, at a slower pace for ApproxSSPS as opposed to the baseline.

(1) Persistent increase in the arrival rate: we first show the capability of ApproxSSPS in cases where the arrival rate increases persistently, up to a factor of five times greater than the processing rate of the system. The latency target in this scenario is 1 s. Results appear in Figure 3.

Both systems, the tree-based baseline [8] and our system, ApproxSSPS, start with no sampling, as they both have the capacity to process the arrived data. We notice that as the arrival rate increases, both systems employ their own controllers to shed the extra data, aiming to remain within the latency targets specified (1 s in this case). As seen in Figure 3, our system ApproxSSPS has higher sampling rates (a desirable feature that improves the accuracy) for all arrival rates when compared to the baseline binary tree-based system. Both systems can achieve the latency target (1 s in this case). However, being able to retain as much data as possible from the arrived stream has a utility in better trading-off the latency for the accuracy. Stated another way, more data guarantees more accuracy. Notice also that the latency of the system, without applying a latency-aware controller, will bring the system to a halt, as it would be unable to process the excess arrived data that far exceeds its capacity. This is specifically clear after the 60 K tuples per second mark is passed, as shown in Figure 3. The stability of the system by applying the latency controller of ApproxSSPS is maintained even with an increase in the data arrival rates that is five times greater than the processing capacity of the system. The same applies to the baseline, which however tends to shed more data, resulting in undesirable tradeoff between the latency and accuracy targets. ApproxSSPS can achieve the latency target by dropping more stream tuples as the arrival rate increases.

**Figure 4:**
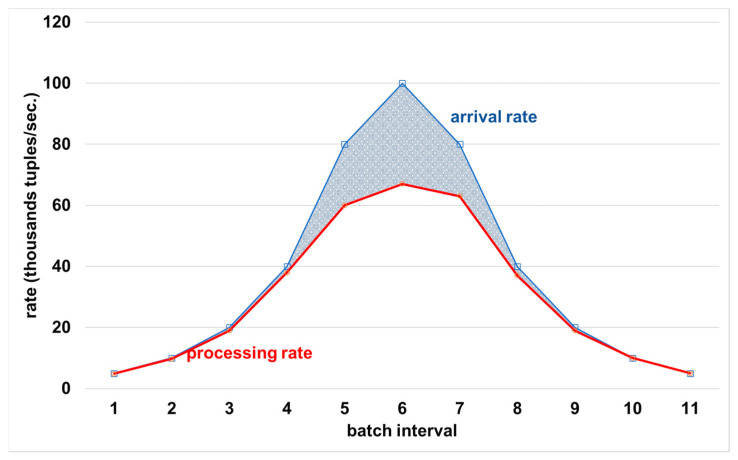
ApproxSSPS ability to catch-up with various oscillations in data stream arrival rates. The patterned area between the two lines shows the dropped tuples. When the area is minimal, it means a higher sampling fraction, which allows more data to continue its way to the approximation operator downstream the system. This leads to a higher accuracy as opposed to those that result in the same load arriving to the baseline system, as shown in Figure 5.

(2) Sudden temporal increase followed by a decrease in arrival rate: Figure 4 shows the behavior of the ApproxSSPS latency controller with fluctuating arrival rates. The arrival rate increases first in a rate that is similar to the processing capacity of ApproxSSPS until batch interval number 3. At that moment, the arrival rate increases gradually, far exceeding the system capacity at batch interval 6. Then it drops slowly until it converges with the processing capacity again at batch interval 9. We can see the discernible pattern, where ApproxSSPS was able to always catch-up with the arrival rate by dropping more data (decreasing the sampling fraction) when the arrival rate exceeded its capacity. The shaded area between the two lines represents the dropped tuples. The processing rate remains stable by dropping data until the arrival rate decreases again to a margin that roughly equals the system capacity. This shows the elasticity of the latency controller of ApproxSSPS and its ability to follow the patterns of the fluctuating arrival rates of data streams.

**Figure 5:**
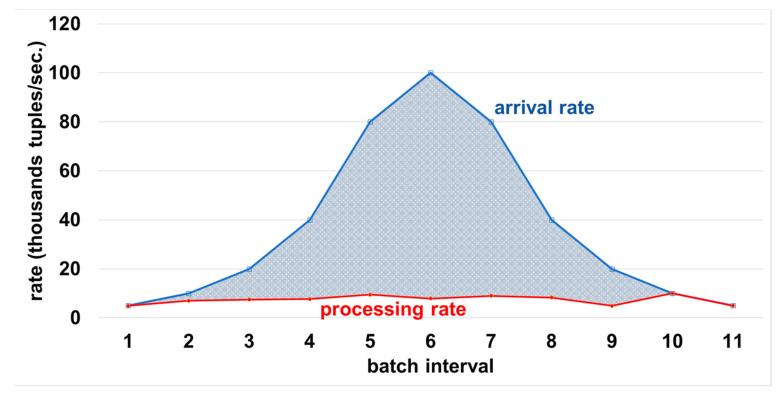
Binary tree-based baseline [8] ability to catch-up with various oscillations in data stream arrival rates. The patterned area between the two lines shows the dropped tuples. More tuples are dropped as compared to ApproxSSPS. The shaded area is significantly greater than the results from applying methods of ApproxSSPS, as shown in Figure 4. This means that a lower sampling fraction, which allows less data to continue its way to the approximation operator downstream the system. This leads to a lower accuracy as opposed to those results with the same load arriving to the ApproxSSPS system, as shown in Figure 4.

To compare the capability of the latency controller of ApproxSSPS with that of the tree-based baseline [8], Figure 5 shows how the baseline behaves with the same data load oscillations. The baseline is also able to catch-up with the oscillation, but drops more data in doing so. This is so, because the baseline has a processing capacity that is lower than that of our system, ApproxSSPS, probably attributable to the fact that the sampling method that they feature (tree-based-like stratification) imposes unnecessarily extra overheads that are not amortized by the benefits of their latency controller.

Both scenarios show that ApproxSSPS is more adept at achieving a plausible balance between the latency/accuracy tradeoff, as it is able to catch-up with varying intensities of data oscillating rates while dropping less data as compared to the baseline.

#### 4.3.2. Accuracy Controller Ability in Trading off QoS Goals

In the previous tests, we concentrated on the efficiency of the latency controller of ApproxSSPS. Even though ApproxSSPS achieved a more plausible tradeoff with the accuracy target, the focus was on keeping the system stable by achieving the latency target. In this subsection, we focus on the ability of the accuracy controller of ApproxSSPS in fulfilling pre-specified accuracy targets. We use the same continuous spatial query. For Shenzhen data, ‘computing the average speed of mobility data’; for the NYC data, ‘computing the average trip distance travelled by taxicabs’. We depend on two parameter configurations. We first vary the geohash precision (from 25 to 30), while varying the sampling fraction (with step size equal to 20, equivalent to sampling fractions ranging from 20% to 80%). We then measure the standard error that results by using each system independently (ApproxSSPS against the tree-based baseline [8]). Second, we vary the spatial query error bound (margin-of-error) and the confidence level. Then, we measure the sample size that is required to fulfill this accuracy requirement.

**Figure 6:**
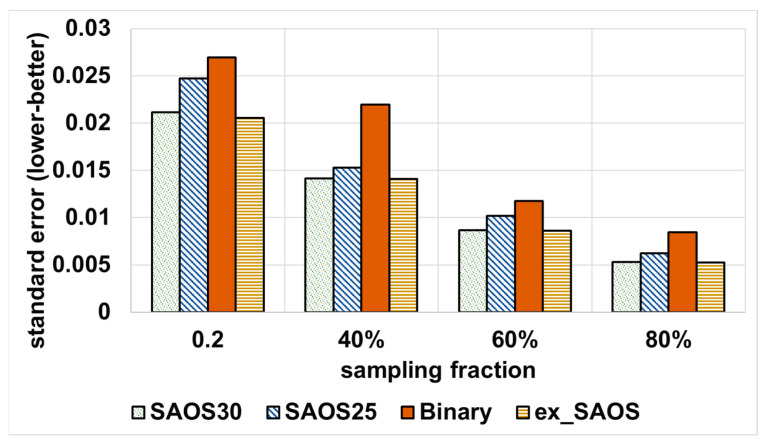
Standard error achieved by all systems, including ApproxSSPS: ‘computing the average speed in Shenzhen mobility data’. Generally, ex-SAOS and SAOS at geohash precision 30 achieve the lowest standard error. SAOS at geohash 25, instead, achieves a higher standard error, which, however, remains lower than that of the baseline.

(1) Varying the geohash for SAOS (25 and 30) and varying the sampling fraction for SAOS and ex-SAOS, we compare the achieved standard error of ApproxSSPS (by employing SAOS and ex-SAOS within the *sampler*) against the baseline (which applies a binary tree stratification sampler). Figure 6 shows the results we obtained for Shenzhen data.

**Figure 7:**
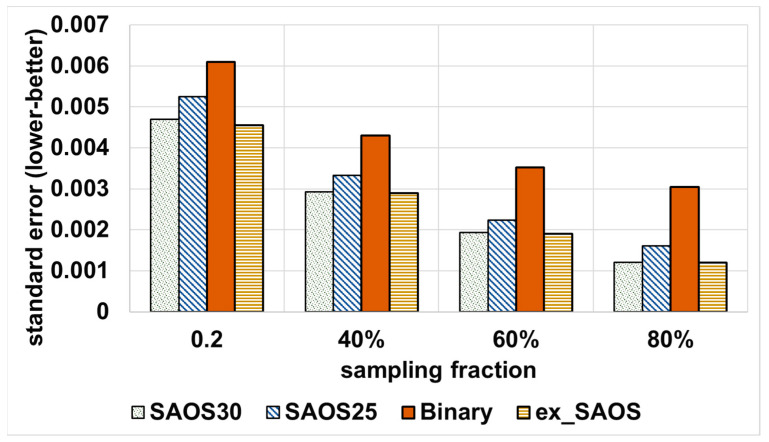
Standard error achieved by all systems, including ApproxSSPS: ‘computing the average “trip distance” in NYC taxicab mobility data’. Similar to Shenzhen data, ex-SAOS and SAOS at geohash precision 30 achieve the lowest standard error. SAOS at geohash 25, instead, achieves a higher standard error, which, however, remains significantly lower than that of the baseline.

As compared to the tree-based baseline, Figure 6 shows that we obtain a lower SE for all sampling fractions when employing SAOS and ex-SAOS incorporated with the sampler of ApproxSSPS. Notice, however, that for both systems, with more sampled data, we obtained a lower SE, but ApproxSSPS always outperformed the tree-based baseline by statistically plausible margins. The same trend occurs with the NYC taxicab dataset, as shown in Figure 7.

**Figure 8:**
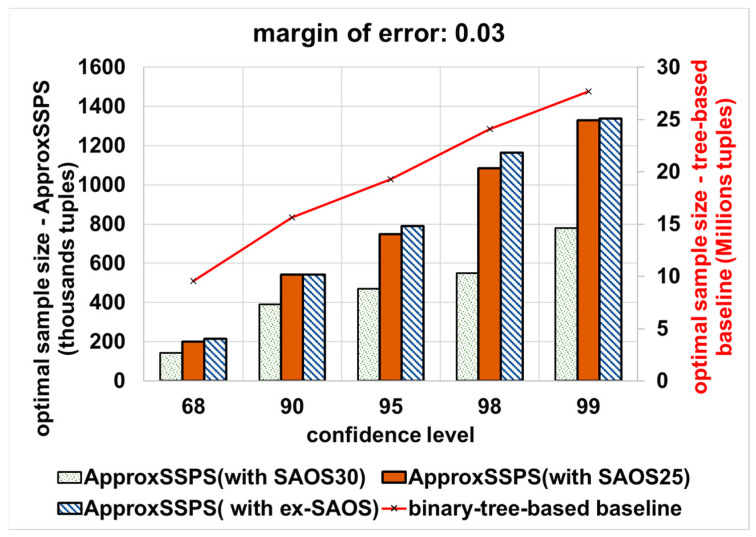
The optimal sample size that is required to achieve a stringent accuracy target (an error bound that equals 0.03): “computing the average ‘speed’ in Shenzhen mobility data”. ApproxSSPS with all geohash precisions and for all confidence levels achieves a significantly much lower optimal sample size as opposed to the baseline. It is a discernible pattern that all methods require a higher sample size as the confidence level increases.

(2) Varying the error bound (margin-of-error) and the confidence level: we vary the error bound (from a stringent 0.03 error bound to a more permissive error bound that equals 0.09) and the confidence level (most common are 68%, 90%, 95%, 98%, and 99%). Then we wait until all data are consumed for every confidence level and error bound combination (pair). Thereafter, we calculate the optimal sample size required to achieve the desired error bound and confidence level pairs. Figure 8 shows the results for an accuracy target that is equal to 0.03, where with ApproxSSPS (depending on either SAOS or ex-SAOS in the *sampler* of ApproxSSPS) we obtain a minimal sample size compared to the binary tree baseline. Notice that, since the baseline applies the Hoeffding inequality, it requires an elevated sample size on the order of millions (the y-axis on the right), while on the other hand, for our system, ApproxSSPS, we require much less data sample size. This is attributable to the fact that, with Hoeffding inequality, there is the squared difference between the max and min values of the target variable, thus significantly increasing the sample size needed. In this case, on average, the accuracy controller of ApproxSSPS (while featuring SAOS with a geohash precision 30) needs 98% less data as compared to the tree-based baseline. Moreover, ApproxSSPS needs, on average, 96.2% (featuring SAOS with geohash precision 25 in its sampler), and 96.1% (featuring ex-SAOS in its sampler) less tuples as compared to those needed by the baseline.

**Figure 9:**
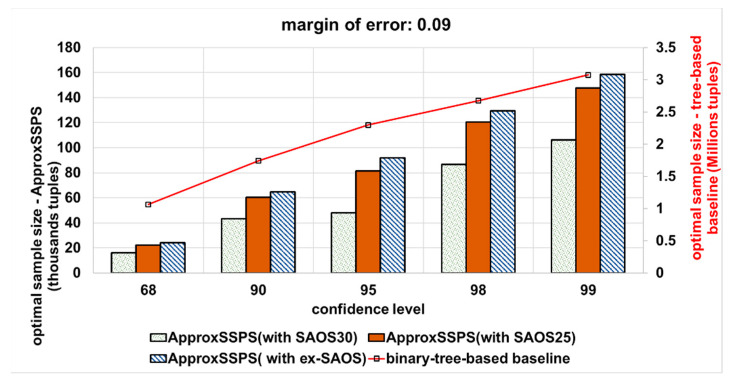
The optimal sample size that is required to achieve a more permissive accuracy target (an error bound that equals 0.09) for both systems (ApproxSSPS and the tree-based baseline): “computing the average ‘speed’ in Shenzhen mobility data”. ApproxSSPS with all geohash precisions and for all confidence levels achieves a significantly much lower optimal sample size as opposed to the baseline. It is a discernible pattern that all methods require a higher sample size as the confidence level increases.

The same pattern occurs for a more permissive accuracy target (e.g., an error bound that equals 0.09) as it is shown in Figure 9. We refer to a higher error bound as ‘more permissive’, because it results in dropping more tuples while fulfilling the target accuracy. This has a direct plausible effect on the latency. In this case, on average, the accuracy controller of ApproxSSPS (while featuring SAOS with a geohash precision 30) needs 97% less data as compared to the tree-based baseline. ApproxSSPS also needs around 96.3% (featuring SAOS with geohash precision 25 in its sampler) and 96% (featuring ex-SAOS in its sampler) less tuples as compared to those needed by the baseline.

**Figure 10:**
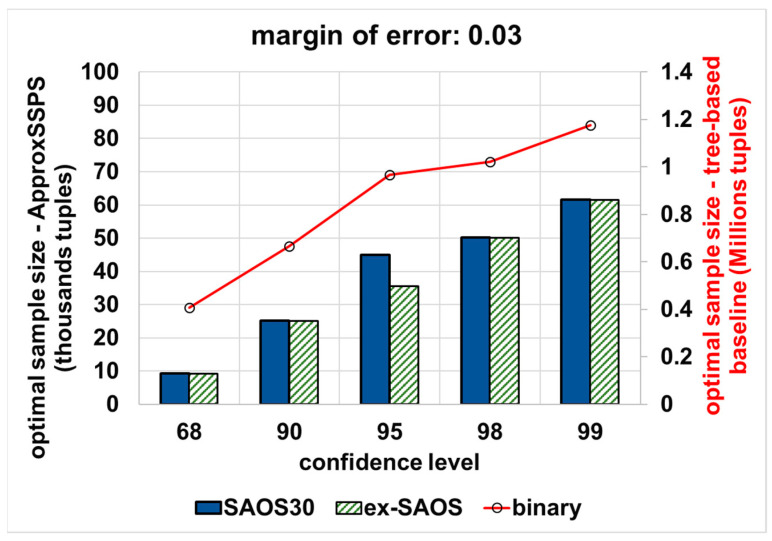
The optimal sample size that is required to achieve a stringent accuracy target (an error bound that equals 0.03): “computing the average ‘trip distance’ in NYC taxicabs mobility data”. ApproxSSPS with all geohash precisions and for all confidence levels achieves a significantly much lower optimal sample size as opposed to the baseline. It is, however, a discernible pattern that all methods require a higher sample size as the confidence level increases.

**Figure 11:**
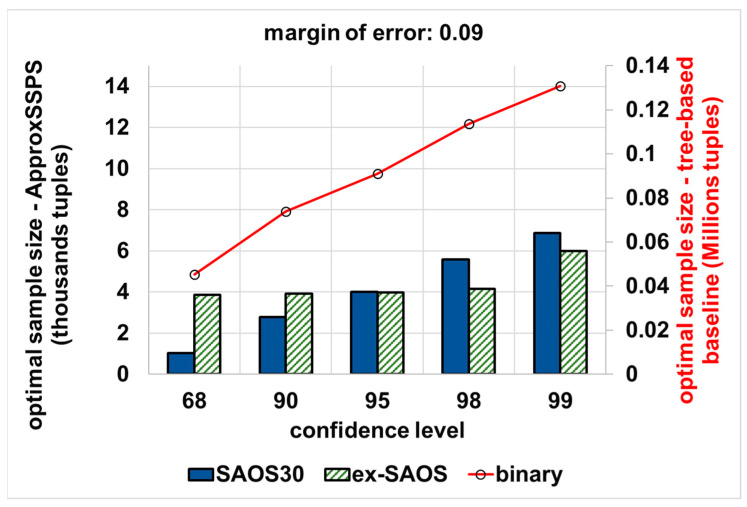
The optimal sample size that is required to achieve a more permissive accuracy target (an error bound that equals 0.09) for both systems (ApproxSSPS and the tree-based baseline): “computing the average ‘trip distance’ in NYC taxicabs mobility data”. ApproxSSPS with all geohash precisions and for all confidence levels achieves a significantly much lower optimal sample size as opposed to the baseline. It is a discernible pattern that all methods require a higher sample size as the confidence level increases.

Similar results are obtained on the NYC data as shown in Figure 10 and Figure 11. In the case of an error bound that equals 0.03, on average, the accuracy controller of ApproxSSPS (while featuring SAOS with a geohash precision 30) needs 60.9% less data as compared to the tree-based baseline. ApproxSSPS also needs around 63% (featuring ex-SAOS in its sampler) less tuples as compared to those needed by the baseline.

In case of an error bound that equals 0.09, on average, the accuracy controller of ApproxSSPS (while featuring SAOS with a geohash precision 30) needs 99.5% less data as compared to the tree-based baseline. ApproxSSPS also needs around 99.4% (featuring ex-SAOS in its sampler) less tuples as compared to those needed by the baseline.

In a sense, there would always be a tradeoff between accuracy and latency. Recall that QoS goals related to those would be of higher accuracy and lower-latency. Since ApproxSSPS features SAOS and es-SAOS in its spatial-aware *sampler*, we have shown that more gain in the latency deterministically means more loss in the accuracy. However, those figures should be controllable in a way that satisfies the best possible combination.

As controversial as that may sound, we undoubtedly know that we obtain a lower latency by operating on less data, but less data means less accuracy. We notice that less data does not always mean casting away very far from the desired error bound, because this highly depends on our conception of things. In other terms, the less the confidence level the user is seeking, the less the data that are required to achieve that level without surpassing the error bounds. This leads to achieving more gain in the latency; thereby a better plausible balance between the contradicting QoS goals (high accuracy versus low latency) is achieved by the controllers of ApproxSSPS.

### 4.4. Results Summary and Discussion

Our results discussed in Section 4.3 communicate several important findings that should be considered carefully when designing future SPEs for processing IoT data in smart cities. First, IoT data shapes should be treated as a priority, in every aspect of the design, and for all methods involved within the processing pipeline. For example, the spatial dimensions of data require application of appropriate spatial indexing methods, such as multidimensionality reduction approaches (e.g., geohashes). This allows higher accuracy, while minimizing latency in processing large amounts of fast-arriving IoT data streams. Moreover, it enables the seamless application of AQP methods, such as spatial sampling in the context of smart cities. Our results prove that spatial sampling methods that are specifically oriented toward IoT smart city data improve the overall performance of the system, in terms of achieving time-based and accuracy-based QoS goals. On the contrary, general-purpose data sampling methods risk causing a high loss in the accuracy of the results, as with the case of the baseline discussed in Section 4.1. The baseline opts for a complex stratified-like method that does not consider the characteristics of data shapes (i.e., spatial, temporal, etc.). In Section 4.3, we showed that this leads to selecting samples that are not representative (in the context of smart cities). Second, any method introduced to the SPE for QoS control should consider the extra overhead caused by the additional layers built atop the base system. In ApproxSSPS, we chose to add QoS controllers that are simple, yet appealing, and do not add discernible overheads to the base system. This enabled ApproxSSPS to remain stable during various fluctuations in data stream skewness and arrival rates. On the contrary, the binary tree-based baseline opted for more complex QoS-aware options. In Section 4.3, we showed that this adds a significant overhead to the system and can bring the system to a halt, in some cases; thus, counteracting the benefits that should be gained through the introduction of time-based and accuracy-based QoS controllers.

## 5. Conclusions

Real-time analytics of IoT mobility data are essential in today’s dynamic smart cities. Data stream processing systems receive large amount of geo-referenced mobility data and are required to obtain accurate insightful analytics in a timely fashion. However, due to the temporal intensity and skewness of the arrival data rates, current systems, in their stock versions, are unable to meet pre-specified QoS goals [2,7]. In this paper, we show the design and implementation of ApproxSSPS, supporting highly efficient spatial AQP over Spark Structured Streaming. ApproxSSPS feature controllers work synergistically to achieve those targets. Using real geo-referenced mobility data from IoT in smart cities, we show the efficiency of ApproxSSPS in achieving the desired latency or/and accuracy QoS goals. In addition to its ability to strike a plausible balance whenever high conflict between accuracy and latency occurs, we implemented ApproxSSPS on Apache Spark; it is also portable to other stream processing systems, such as Storm [28], Flink [5], Apache Kafka streams [29], and STREAM [30]. As a future research perspective, we are exploring the opportunities of extending the features we provide in ApproxSSPS, so that they reach other, more complex smart city workloads (for example, being able to sample two geo-referenced data streams and join them for more complex analytics). We plan to add a support that enables joining a geo-referenced data stream containing meteorological data (e.g., temperature, humidity etc.,) with another geo-referenced mobility data stream (taxicab mobility trajectory data) for more advanced analytics. For example, to enable answering interesting queries, such as: “what are the Top-3 neighborhoods in the Shenzhen city in China, in terms of mobility traffic, with a ‘temperature’ that is, on average, greater than 20 degrees”.

At the same time, ApproxSSPS still exhibits few limitations. First, it supports a limited set of aggregate geo-statistics (e.g., count, average, and sum) that are predefined within the *approximator* component. Those aggregate functions are applied to the samples drawn using the sampler component (which employs either SAOS or ex-SAOS). Future research will include enabling a hot-swappable component that allows end-users to seamlessly plug more advanced and custom statistical functions (e.g., advanced aggregate functions) so that they apply to the data coming from the sampler, as part of the data processing pipeline. In addition, the sampler component can be migrated to IoT devices instead of the cloud, so that we port the sampling part to the IoT near the data. By doing that, we relieve some pressure from the system in such a way that the system will only then be responsible for the aggregate computations instead of having to also deal with another task, such as sampling. Moreover, ApproxSSPS currently is oriented to spatial data with little awareness of the temporal dimension. A future plan is to extend it so that it fully supports the temporal dimension in addition to the spatial dimension. This is planned so that we incorporate a spatiotemporal index that can be plugged to the sampler, in order to enable the sampler-to-sample representative data from fast-arriving data streams, considering both temporal and spatial dimensions. Moreover, we plan to extend the architecture, so that it enables sampling data from multiple data streams and join them on the spatial and/or temporal dimensions. For example, joining mobility data with geo-referenced meteorological data to answer, interactively, more advanced aggregate queries, such as the following: “what are the neighborhoods in Rome in Italy with [the] most PM2.5 concentrations caused by unprecedented vehicle mobility traffic during each month of a year”.

## Figures and Tables

**Table 1:** z_α/2_ values for various confidence levels in normal distributions.

Confidence Level	z_α/2_
68%	1
90%	1.645
95%	1.96
98%	2.326
99%	2.576

## Data Availability

Publicly available datasets were analyzed in this study. NYC taxicabs dataset can be found here: https://www1.nyc.gov/site/tlc/about/tlc-trip-record-data.page. Accessed on: 5 January 2021.
